# Ultrafast Red Light Activation of *Synechocystis* Phytochrome Cph1 Triggers Major Structural Change to Form the Pfr Signalling-Competent State

**DOI:** 10.1371/journal.pone.0052418

**Published:** 2012-12-26

**Authors:** Derren J. Heyes, Basile Khara, Michiyo Sakuma, Samantha J. O. Hardman, Ronan O'Cualain, Stephen E. J. Rigby, Nigel S. Scrutton

**Affiliations:** Manchester Institute of Biotechnology and Photon Science Institute, Faculty of Life Sciences, University of Manchester, Manchester, United Kingdom; University of North Dakota, United States of America

## Abstract

Phytochromes are dimeric photoreceptors that regulate a range of responses in plants and microorganisms through interconversion of red light-absorbing (Pr) and far-red light-absorbing (Pfr) states. Photoconversion between these states is initiated by light-driven isomerization of a bilin cofactor, which triggers protein structural change. The extent of this change, and how light-driven structural changes in the N-terminal photosensory region are transmitted to the C-terminal regulatory domain to initiate the signalling cascade, is unknown. We have used pulsed electron-electron double resonance (PELDOR) spectroscopy to identify multiple structural transitions in a phytochrome from *Synechocystis* sp. PCC6803 (Cph1) by measuring distances between nitroxide labels introduced into the protein. We show that monomers in the Cph1 dimer are aligned in a parallel ‘head-to-head’ arrangement and that photoconversion between the Pr and Pfr forms involves conformational change in both the N- and C-terminal domains of the protein. Cryo-trapping and kinetic measurements were used to probe the extent and temporal properties of protein motions for individual steps during photoconversion of Cph1. Formation of the primary photoproduct Lumi-R is not affected by changes in solvent viscosity and dielectric constant. Lumi-R formation occurs at cryogenic temperatures, consistent with their being no major structural reorganization of Cph1 during primary photoproduct formation. All remaining steps in the formation of the Pfr state are affected by solvent viscosity and dielectric constant and occur only at elevated temperatures, implying involvement of a series of long-range solvent-coupled conformational changes in Cph1. We show that signalling is achieved through ultrafast photoisomerization where localized structural change in the GAF domain is transmitted and amplified to cause larger-scale and slower conformational change in the PHY and histidine kinase domains. This hierarchy of timescales and extent of structural change orientates the histidine kinase domain to elicit the desired light-activated biological response.

## Introduction

**Figure 1:**
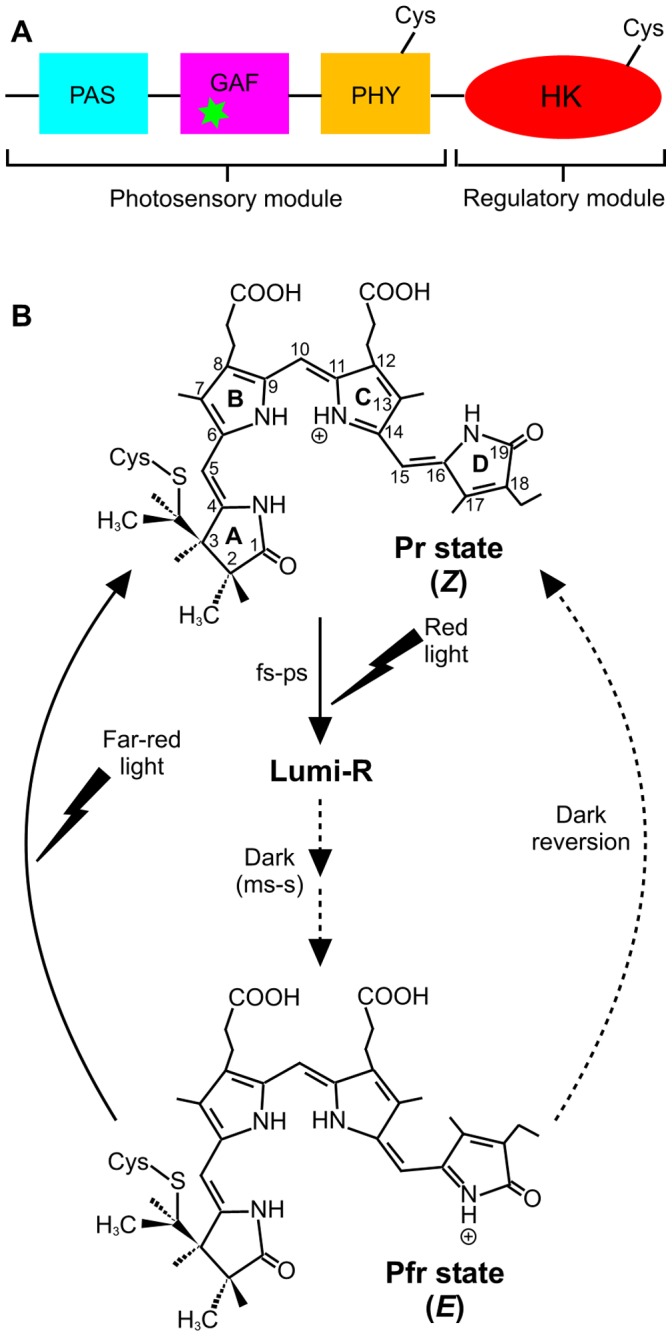
Domain architecture and reaction scheme of Cph1. (A). Domain architecture of phytochrome, consisting of an N-terminal photosensory module that comprises the PAS, GAF and PHY domains, and a C-terminal regulatory module that contains a histidine-kinase like domain (HK) [1]. The Cys residues that have been used for spin-labeling studies in the present work are shown and the green asterisk indicates the phycocyanobilin cofactor. (B). The proposed photoconversion of the Pr to Pfr states of the phycocyanobilin cofactor in Cph1 phytochrome. The chromophore in the Pr state is shown as *ZZZ*ssa at the AB, BC, and CD rings, respectively [5]. Illumination with red light triggers photoisomerization about the C15–C16 methine bridge to give the primary photoproduct, Lumi-R, which is subsequently converted to Pfr in several light-independent steps on the millisecond-to-second timescale. The Pr state can be regenerated from Pfr by excitation with far-red light or by a slow dark reversion process [1].

Phytochromes are photoreceptor proteins that regulate a number of red and far red light signalling responses in plants and microorganisms [1], [2]. In plants they are a major class of photoreceptors required for adaptation to diurnal and seasonal light fluctuations, and plant adaptation to agricultural environments will depend on better understanding of phytochrome function at the molecular level. This molecular picture is beginning to emerge through the recent determination of three-dimensional structures for related microbial phytochromes [3]–[8]. Prokaryotic phytochromes are homodimers that have complex domain architecture and contain a bilin (linear tetrapyrrole) cofactor as the chromophore (Figure 1A). An N-terminal photosensory module contains three domains, including Per/Arndt/Sim (PAS), cGMP phosphodiesterase/adenyl cyclase/FhlA (GAF) and phytochrome (PHY) domains [1], [2]. Phycocyanobilin (or phytochromobilin in plants) is attached to the GAF domain by a thioether linkage and defines the unique spectral properties of phytochromes [1], [2], [9]. A histidine kinase (HK) domain forms the C-terminal regulatory module, which corresponds to a serine/threonine kinase (S/TK) domain found in plant phytochromes. The presence of HK and S/TK domains suggests that kinase activity is associated with the signal output of phytochromes [10].

Phytochrome-mediated signalling is initiated by photoconversion of an inactive Pr state to an active Pfr state (Figure 1B). Interconversion between the Pr and Pfr states is triggered by red light (Pr) and far-red light (Pfr) absorption [1]. However, the mechanism of photoconversion is poorly understood, particularly in relation to the thermally-driven conformational changes that follow initial photoactivation, and it is also unclear how photoconversion to the Pfr state affects the C-terminal regulatory domain and how this initiates signal transmission. Spectroscopic studies have suggested that the Pr → Pfr conversion involves a *Z* to *E* photoisomerization of the double bond of the C15–C16 methine bridge between the C and D pyrrole rings of the bilin cofactor [11]–[14]. A recent NMR study of a phytochrome from the thermophilic cyanobacterium *Synechococcus* sp. OS B’, however, suggests photoisomerization of the bilin C4–C5 double bond [15]. The initial photoisomerization of the bilin cofactor is proposed to occur on a picosecond timescale and results in the formation of a primary photoproduct, termed the Lumi-R state, which has previously been shown to adopt the 15*E*a configuration [14], [16]–[21] (Figure 1B). The initial photoisomerization of the bilin cofactor is then followed by a number of slower steps on the microsecond to millisecond timescale to form the Pfr state of the protein (Figure 1B). These are proposed to involve a series of conformational changes in the protein [15] that are linked to deprotonation/reprotonation of the chromophore [22], and ultimately lead to an active, signalling conformation of the C-terminal region.

Currently, the extent of any conformational movements during photoconversion is unclear. The structures of the bilin-binding photosensory modules of several phytochromes have been determined for the Pr state [3]–[7], but there is only limited structural information available for the Pfr state [8], [15]. In the Pr state, the dimer interface is provided by adjacent GAF domains derived from each protein subunit. The PAS and GAF domains are connected by a figure-of-eight knot and a hairpin projection from the PHY domain helps to seal the chromophore pocket, located in the GAF domain, from the solvent. The bilin cofactor is covalently bound to the GAF domain at ring A with additional steric interactions and hydrogen bonds contributed by protein residues to the pyrrole rings and propionate side-chains [3]–[8]. A recent NMR structure of the Pfr state of the GAF domain of a cyanobacterial phytochrome (Cph2) indicates that a series of reversible conformational movements occur within the bilin-binding pocket following photoconversion, which affect intra- and interdomain contacts within the phytochrome dimer [15]. A cryo-electron microscopy structure for full-length *D. radiodurans* phytochrome reveals an extensive helical dimer interface and head-to-head packing of the subunits [23]. This contrasts with head-to-tail packing observed in the crystal structure of the *Synechocystis* phytochrome photosensory module mediated by the equivalent helical interface [5]. However, crystal structures for a full-length phytochrome protein remain elusive and it is unknown how structural change in the photosensory module is transmitted to the C-terminal regulatory module to initiate signalling. Therefore, mapping of the spatio-temporal properties of the structural transitions following the initial photochemical event is crucial to our understanding of phytochrome function at the molecular level.

We have analyzed protein motions required for photoconversion of the Pr state to the Pfr state of the full-length Cph1 phytochrome from *Synechocystis* sp. PCC6803. Site-directed spin labelling (SDSL) and pulsed electron electron double resonance (PELDOR) spectroscopy was used to analyze Cph1 structure in the Pr and Pfr states, and also a truncated form containing only the photosensory module. This allowed us to observe multiple structural transitions by measuring distances between nitroxide spin labels introduced into the target protein. Distance constraints obtained from the SDSL-PELDOR data were used to discriminate between the head-to-head and head-to-tail models derived from existing crystallographic and cryo-electron microscopy studies. We also report cryogenic absorbance spectroscopy and time-resolved spectroscopy studies on the picoseconds to seconds timescale and show that the initial formation of the Lumi-R intermediate is not coupled to a network of long range motions in the protein. We also show that a series of relatively slow conformational changes, which are coupled to the solvent dynamics, are required to attain the final Pfr following fast initial photoisomerization of the phycocyanobilin cofactor.

## Results

### PELDOR Evidence for Photoconversion-linked Conformation Changes in Cph1

PELDOR spectroscopy measurements were used to analyze structural differences between the Pr and Pfr states of *Synechocystis* PCC 6803 Cph1 by measuring distances between nitroxide labels introduced on the surface of the protein. It has been shown previously using other protein systems that at the low temperatures used in PELDOR studies a series of inter-label distances can be observed. These indicate that a distribution of protein conformers exists at low temperature, each conformer representing a minimum in the conformational energy landscape [24]. These complex PELDOR-distance profiles illustrate the potential for observing multiple conformational states in dynamic protein systems using the PELDOR method.

Nitroxide labels were introduced into the Cph1 proteins using an established SDSL protocol [25], by attachment to cysteine residues (either native or engineered) via disulphide bond formation. Spin labels were attached to the native Cys371 of the full-length Cph1 protein and the equivalent position in the N-terminal photosensory region (a truncated form of Cph1). Attachment of the SDSL only at residue Cys371 was confirmed by mass spectral analysis (Figure S1). In addition, spin labels were coupled to the Cph1 variant protein (C371-N733C; see Figure 1A for location of spin labels), so that the spin label was attached to both residues Cys371 (native residue) and Cys733 (engineered residue) and the conformational properties of each of these labeled samples were analyzed at low temperatures in the Pr and Pfr states.

**Figure 2:**
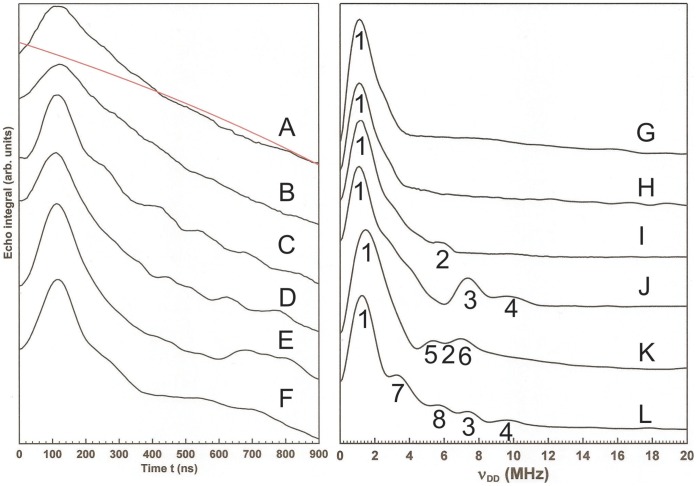
PELDOR analysis of Cph1 conformations. PELDOR data obtained from spin-labeled *Synechocystis* PCC 6803 Cph1. Raw four-pulse ELDOR traces together with the third order polynomial function used to baseline the data (in red) are shown in the left hand panel, while the right hand panel shows the conjugate Fourier transforms of these data. (A) and (G). Pr form of the N-terminal photosensory region with spin-label at C371. (B) and (H). Pfr form of the N-terminal photosensory region with spin-label at C371. (C) and (I). Pr form of full-length Cph1 with spin-label at C371. (D) and (J). Pfr form of full-length Cph1 with spin-label at C371. (E) and (K). Pr form of full-length Cph1 with spin-label at C371 and N733C. (F) and (L). Pfr form of full-length Cph1 with spin-label at C371 and N733C. Pulse sequences and data processing are described in Materials and Methods. Numbers in the right hand panel indicate ν_DD_ and distances referred to in the text.

PELDOR spectroscopy was used to measure the dipolar coupling, ν_DD_, between two unpaired electrons. This dipolar coupling exhibits a dependence on the distance between the two electron spins given by Equation 1 [26]:
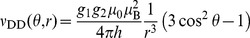
(1)where *g*_1_ and g_2_ are the *g* values of the two spins, *r* is the distance between them, and *θ* is the angle between the inter-spin vector and the applied magnetic field. In order to employ the four-pulse ELDOR technique it is necessary to first estimate some parameters governing the behavior of the electron spins. Two pulse echo decay experiments shown in Figure S2 (supplementary information) allow for the determination of the electron T_M_, which governs the rate of decay of the spin echo, as 573 ns at 30 K. For the double labeled C371-N733C protein the echo decays are best fit by a double exponential with T_M_ 570 ns and 462 ns. From these experiments sufficient signal remains after 1 µs to allow for the measurement of an echo in the absence of nuclear modulation and the estimation of any nuclear modulation frequencies exhibited by the echo. The nuclear modulations can perturb the modulations due to the dipolar coupling that we wish to measure, but they can be effectively eliminated by setting the T time period to 4πn/ω_N_, where n is an integer and ω_N_ is the nuclear modulation frequency (rad s^−1^) [24]. Here T is set to 230 ns. To determine the optimum magnetic fields for the application of pump and detection pulses, echo detected pulse field swept spectra were obtained (Figure S3). The pump pulse was applied at the field corresponding to the intensity maximum, while the detection pulse was applied at the low field turning point, 27 G downfield of the maximum. The raw time evolution data from the four pulse ELDOR experiments, plotting the echo integral variation with time t, are shown in Figure 2 together with the third order polynomial function subtracted as a baseline. The modulation of the echo intensity with time t from the first maximum in baseline corrected data (Figure S4) is given by cos(ν_DD_(t-T)) [24] and thus the modulation frequencies can be extracted from Fourier transformation of data starting from the top of the first maximum (cosine transformation). The Fourier transforms of the baseline-corrected raw data are shown in Figure 2. These data provide estimates of the inter-label distances present within the protein population via measurement of the electron-electron dipolar coupling, ν_DD_, and the use of Equation 1.

Initial PELDOR experiments were performed on the Cys371-labeled N-terminal photosensory region of Cph1, comprising the PAS, GAF and PHY domains, to report on the distance between the Cys residues in each half of the dimeric protein. Only one ν_DD_ value is evident, 1.1 MHz, in the Fourier transform data for both the Pr and Pfr states of the N-terminal photosensory region (shown as feature 1 in Figure 2G and 2H), corresponding to a distance of 36 Å between the spin labels on the two Cys371 residues of the dimer. However, measurements on the Cys371-labeled full-length Cph1 protein, which includes the histidine kinase (HK) region, show that differences exist between the two states of the protein. The Fourier transforms of the raw, baseline corrected data in this instance (Figure 2I and J) show populations of Cph1 conformers in which the inter-label distance is shorter than 36 Å for both forms of the protein. The Pr form exhibits an additional distance of 21 Å (marked as 2) while the Pfr form exhibits two additional distances of 19 Å and 17 Å (marked as 3 and 4). The real (cosine) discreet Fourier transform used here is a linear operator, thus the integrals of the lines in the Fourier transform spectra reflect the echo intensities at t = 0 and these are in turn proportional to the number of spin labels (electrons) giving rise to each line in the Fourier transform. This suggests that the shorter inter-label distances are present as minor components of the conformational distribution in frozen solutions of full-length Cph1.

The double-labelled full length Cph1, which has a second spin label at residue 733 through the N733C mutation, produces the data in Figure 2E, F, K and L. There are several dipolar coupling values evident in addition to those observed in the data from single-labeled protein which we associate with the extra spin label at C733. These correspond to additional distances of 21 Å (marked as 5) and 19 Å (marked as 6) in the Pr form and 25 Å and 21 Å in the Pfr form (marked as 7 and 8, respectively). The intensities of the 2-pulse field swept spectra of the double-labeled protein (Figure S3) suggest that the C733 label is present at only about 40% concentration of the C371 label. Note that the possibility that small differences in inter-label distance arise from a change in the conformational distribution of the label, rather than of the domain to which it is attached, cannot be excluded. However, such an effect must then arise from a change in the local protein structure around the labels (i.e. around residues 371 and 733), which must in itself be linked to the photoisomerization event. The larger changes in inter-label distance (those greater than 10 Å) are unlikely to be due to such effects since it is known from X-ray crystal structures that the labels are attached to residues within stable multi-strand β-sheet structures displaying close packing of amino acid side chains [5], [27].

### Cryo-trapping of Intermediate States in the Cph1 Photocycle

**Figure 3:**
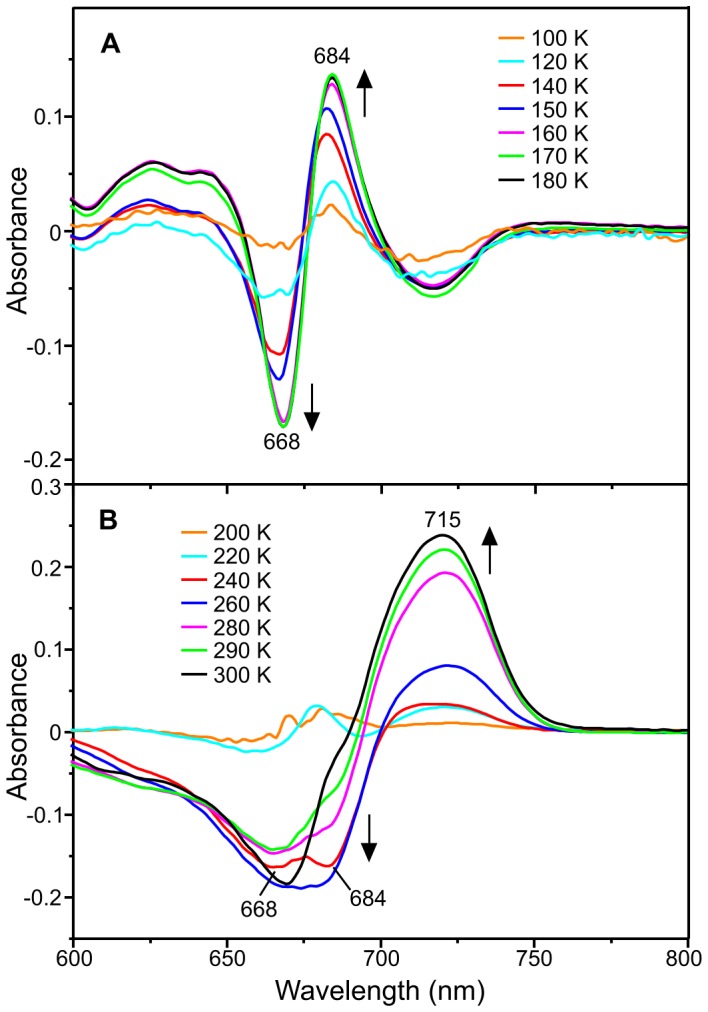
Characterization of the Pr → Pfr conversion by cryogenic absorbance measurements. (A). 77 K absorbance difference spectra of samples containing 15 µM Cph1 after illumination for 10 mins at different temperatures ranging from 77 K to 180 K. The difference spectra were obtained by using a non-illuminated sample as a blank. The formation of the absorbance peak at 684 nm and simultaneous disappearance of the absorbance band at 668 nm at higher temperatures are indicated by the arrows. (B). 77 K absorbance difference spectra of samples containing 15 µM Cph1 after illumination at 180 K for 10 mins and incubation in the dark for 10 mins at increasing temperatures. The difference spectra were obtained by using the sample that was illuminated at 180 K as a blank. The arrows indicate the formation and disappearance of the different absorbance bands at higher temperatures. The raw absorbance spectra are shown in figure S4.

**Figure 4:**
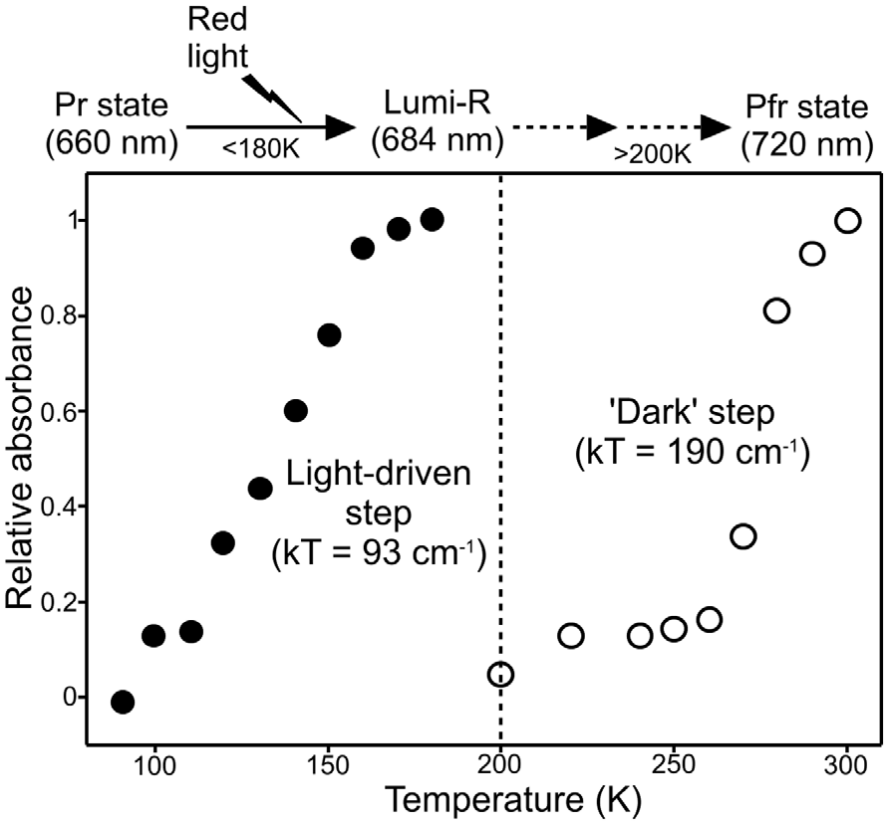
The temperature dependence of the steps involved in the Pr → Pfr photoconversion. The temperature dependence of the initial formation of the Lumi-R state (•) and the remaining step(s) to form the Pfr state (○) were obtained by analysis of the cryogenic absorbance measurements (Figure S5) and are shown together with the kT value for each step, where k is the Boltzmann constant (0.695 cm^−1^) and T is the mid-point temperature of each process. The ‘glass transition’ temperature of proteins is also shown as a reference (dashed line).

The energetic or thermal barriers to these conformational changes were investigated by monitoring the initial light-induced step in the photoconversion of the Pr to Pfr state over a range of temperatures. Absorbance spectra, recorded at 77 K after illuminating samples with red light at the desired temperature for 10 mins, show that an initial light-dependent reaction results in formation of the Lumi-R intermediate at temperatures below 200 K (Figures 3A and S5). This step involves a decrease in the absorbance band at 668 nm together with the simultaneous appearance of a new intermediate absorbing at 684 nm (Figure 3A). The temperature dependence of this photochemical step was obtained by plotting the absorbance at 684 nm against the temperature of illumination and it reveals that completion was reached at temperatures below 180 K (Figures 4 and S6).

In order to identify additional conformational changes occurring on forming of the Pfr state, the Pr form of Cph1 was firstly illuminated with red light (180 K for 10 min) to form the Lumi-R state. These samples were then warmed to progressively higher temperatures in the dark for 10 mins and the non-photochemical steps were analyzed by measuring absorbance spectra at 77 K (Figures 3B and S5). A further decrease in the main Pr absorbance band at approximately 668 nm can be observed at temperatures above 200 K together with disappearance of the Lumi-R absorbance band at 684 nm (Figure 3B). This is accompanied by a simultaneous increase in the absorbance peak at 715 nm, which represents the Pfr state of the protein. However, it should be noted that there is no clear, single isosbestic point in these absorbance difference spectra. Plotting the increase in absorbance at 715 nm as a function of temperature, reveals that formation of the final Pfr state can only proceed at higher temperatures and requires incubation at room temperature (Figures 4 and S6).

### Solvent-coupled Protein Motions are not Required for the Initial Photoisomerization Dynamics

**Figure 5:**
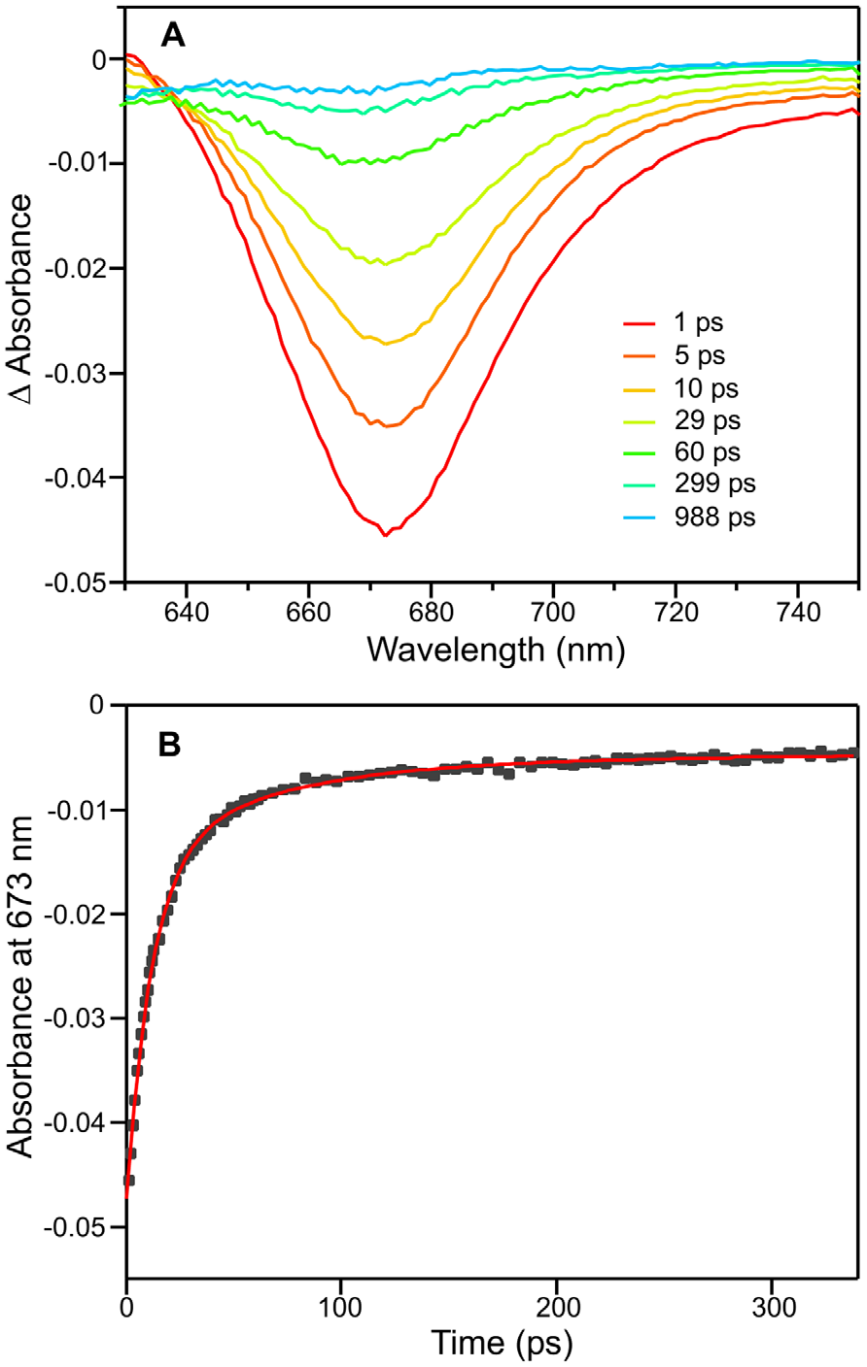
Characterization of the initial photoisomerization dynamics of the Pr state of Cph1. Cph1 was contained in a 5% sucrose solution and absorbance spectra recorded after photoexcitation with a laser pulse centred at ∼590 nm. (A) Transient absorption difference spectra at delay times of 1, 5, 10, 29, 60, 299, and 988 ps after excitation. (B) Kinetic transient at 673 nm (black squares) with a fit of the data to 2 exponentials shown in red.

The kinetics of the Pr to Pfr photoconversion were monitored using a combination of ultrafast pump-probe spectroscopy and laser flash photolysis over a range of sucrose and glycerol concentrations. The initial photoisomerization dynamics were studied using transient pump-probe measurements over 1 ns following excitation with a laser pulse at 580 nm (Figure 5A). The transient absorption difference spectra show identical spectral features to those reported previously [17] with a strong bleach centred at 670 nm, due to a combination of the ground-state bleach and stimulated emission, and a weak transient (positive) feature below 640 nm that is assigned to excited-state absorption. The recovery of the ground-state bleach, the stimulated emission and the decay of the excited-state absorption all exhibit identical kinetics [17] and therefore, the kinetic analysis of the data was performed at a single probe wavelength of 673 nm (Figure 5B). The kinetic data were fitted to 2 exponentials with average lifetimes of 12.0 and 68.9 ps (Table 1), which are in good agreement with previously observed values of 15.1 and 97 ps [17] and 14 and 134 ps [18]. It should also be noted that the relative amplitude of the faster phase is significantly larger (approximately 75%) than it is for the slower phase. Moreover, the initial photoisomerization dynamics show no measurable difference in the lifetime values at higher sucrose and glycerol concentrations (Table 1), suggesting that this step is not dependent on solvent viscosity.

**Table 1:** The effect of solvent viscosity (η) on the lifetimes associated with the initial photoisomerization dynamics of the Pr state of Cph1.

Sample	η (cP)	τ1 (ps)	Relative amplitude	τ2 (ps)	Relative amplitude
5% sucrose	1.14	12.0±0.3	77%	68.9±6.1	23%
10% sucrose	1.33	12.1±0.4	79%	65.0±7.4	21%
30% glycerol	2.74	12.6±0.7	81%	62.8±13.4	19%
30% sucrose	3.18	10.4±0.5	70%	39.8±3.5	30%
60% sucrose	58.4	11.0±0.4	72%	42.7±3.4	28%

The lifetime values were obtained by fitting the decay at 673 nm, recorded over 350 ps (Figure 5B), to 2 exponentials.

### Solvent-coupled Protein Motions are Required for Slower Conformational Changes

**Figure 6:**
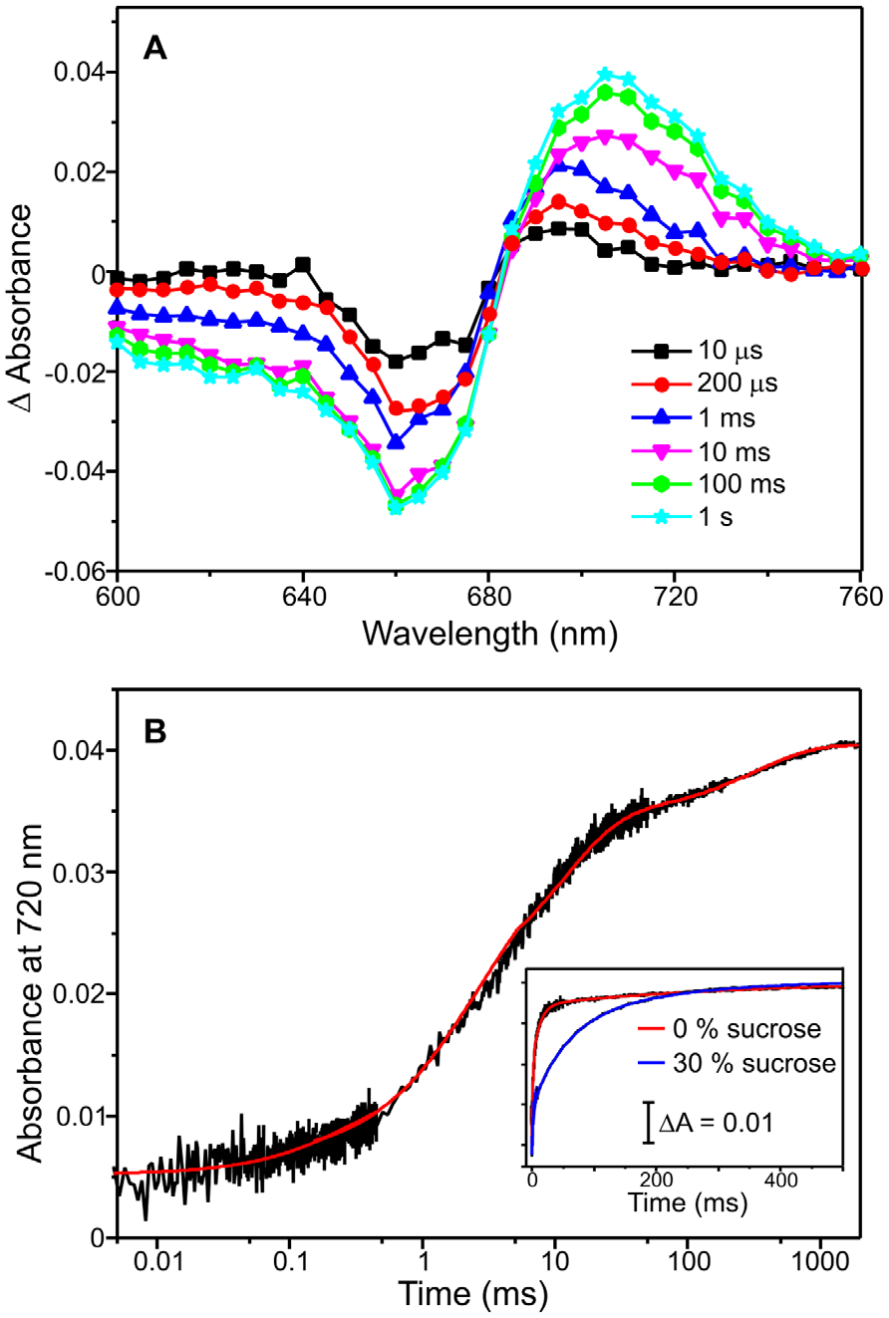
Characterization of the Pr → Pfr conversion by laser photoexcitaion measurements. (A) Absorbance difference (‘action’) spectra following photoexcitation of 15 µM Cph1 (Pr form) with a 6 ns laser pulse at 660 nm. The spectra were created by measuring the absorbance change at the respective wavelength at various timepoints after laser excitation. (B) Typical kinetic traces measured at 720 nm over 1 second on a logarithmic timescale following photoexcitation of 15 µM Cph1 (Pr form) with a 6 ns laser pulse at 660 nm. The data were fitted to 4 kinetic phases to obtain rate constants (red line). The inset shows kinetic transients at 720 nm at 0% sucrose and 30% sucrose to illustrate the effect of solvent viscosity. All traces were collected at 20°C as described in the Materials and Methods section.

The remaining steps in the formation of the Pfr state of the protein were followed by measuring kinetic transients over a range of timescales (µs to second timescale) at different wavelengths after triggering the reaction with a 6 ns laser pulse at 660 nm. Time-dependent difference spectra recorded between 600 nm and 760 nm show that the slower steps in the formation of the Pfr state involve a further decrease in the absorbance band at 660 nm together with a simultaneous, gradual increase in absorbance at ∼720 nm (Figure 6A). Typical transients at 660 nm (Figure S7) and 720 nm (Figure 6B) reveal that 4 kinetic phases are required to fit the data with the relative amplitude of each phase shown in Table 2. The rate constants for each of the 4 steps are approximately 3200 s^−1^, 350 s^−1^, 45 s^−1^ and 2 s^−1^ (Table 2).

**Table 2:** The effect of solvent on the rates of the slower steps in the Pr → Pfr photoconversion.

Step	Relative amplitude	σ (cP)	Rate (s^−1^)			
			H_2_O	5% ethanol	5% isopropanol	35% sucrose
1^st^	17%	1.26±0.29	3235±400	3100±195	3040±265	1365±80
2^nd^	26%	2.89±0.72	368±60	328±47	314±39	205±41
3^rd^	38%	0.77±0.19	45.9±1.8	40.0±2.9	36.6±3.1	16.4±0.8
4^th^	19%	>40	2.22±0.28	2.01±0.22	1.96±0.21	2.02±0.05

**Figure 7:**
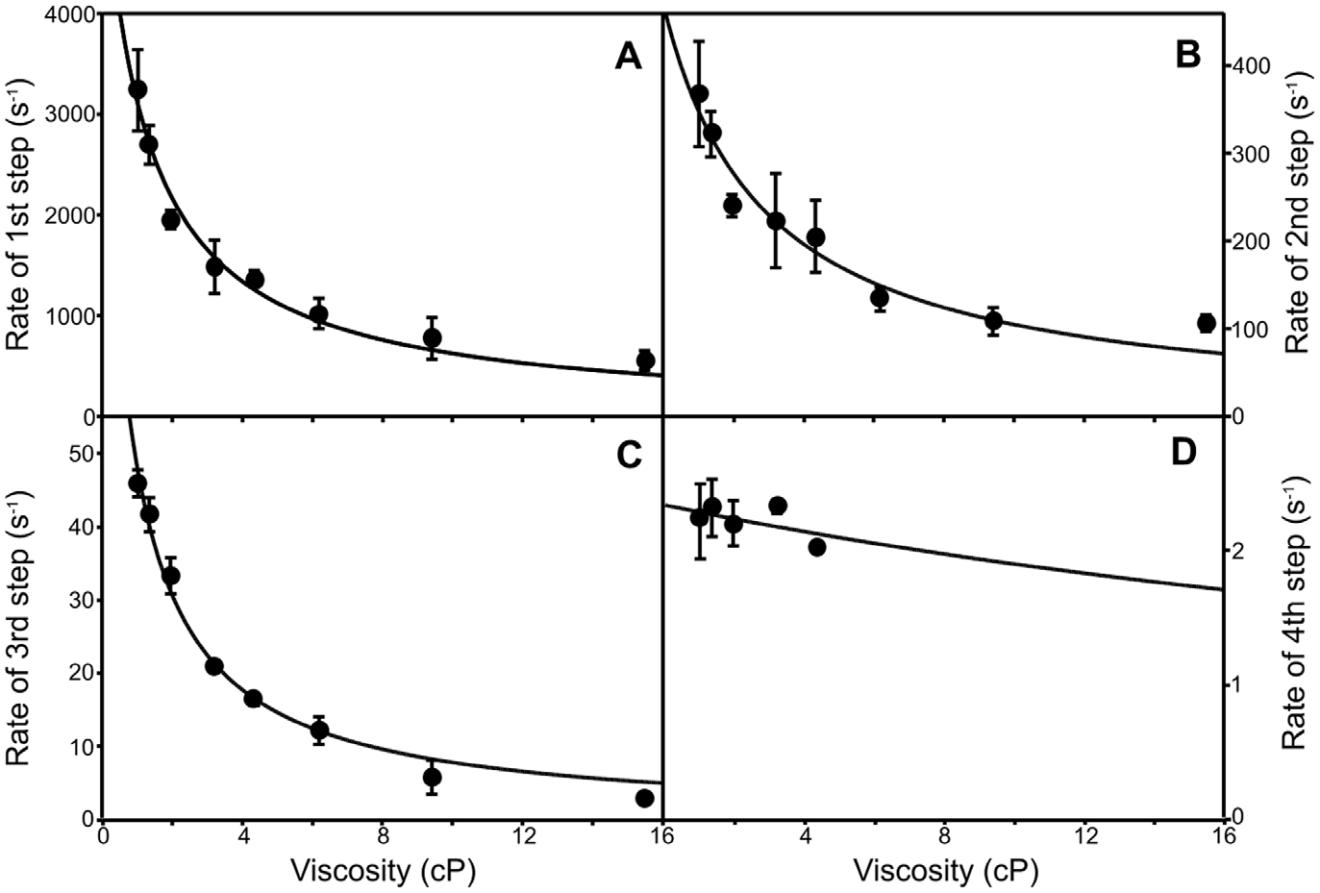
The viscosity-dependence of the slower steps in the Pr → Pfr photoconversion. The viscosity dependence of the rate constant for the 1^st^ (**A**), 2^nd^ (**B**), 3^rd^ (**C**) and 4^th^ steps (**D**) of the increase in absorbance at 720 nm are shown. All measurements were recorded over a range of timescales and the data are fitted to equation 2 as described in the Materials and Methods. The error bars were calculated from the average of at least 3 traces.

The rate and amplitude of each step was measured at 293 K (Materials and Methods). The degree of solvent-coupled dynamics (σ) required for each step were obtained by fitting the viscosity-dependence of the rates to equation 2 (Figure 7).

Measurements were repeated over a range of sucrose and glycerol concentrations to generate viscosity dependencies for each step (Figure 6B inset and 7). The extent of solvent-coupling to each step was determined by fitting the data to equation 2 to assign the contribution of the protein friction (σ) to the total friction of the system (Figure 7 and Table 2). The first three kinetic steps in the conversion of the Lumi-R intermediate to the Pfr state of the protein are influenced by changes in solvent viscosity with the largest effect observed on the third step (Table 1). At higher viscosities (>4 cP) the rate of the third step (Figure 7C) became very similar to the rate of the fourth step; it was therefore not possible to measure an accurate viscosity dependence for the slower step (Figure 7D). To ensure that these effects were not caused by interactions between Cph1 and sucrose, measurements were also conducted using glycerol as viscosogen. Again, rates for the first three steps were found to decrease at higher viscosities (Figure S8). The effect of sucrose and glycerol can also be analyzed in terms of the solvent dielectric as these solvent mixtures possess significantly lower dielectric constants (for example 35% sucrose has ε = 70) compared to water (ε = 78). We investigated if the rate of the slower steps in the reaction was also affected by changes in the solvent dielectric constant (ε), which was altered by the addition of organic solvents to the buffer solution (Table 2). Accordingly, the rate of all 4 steps in the Lumi-R → Pfr reaction are slower in 5% ethanol (ε = 74) and 5% isopropanol (ε = 72) compared to water (ε = 78), although there is only a minor effect on the 4^th^ step. The slower steps in the photoconversion are therefore dependent on both solvent viscosity and the overall dielectric constant of the solvent.

## Discussion

The mechanism by which phytochromes sense red light for signalling is still unclear. In particular, it is unknown how structural change in the photosensory region of the protein, which accompanies conversion of the Pr to Pfr states on absorption of red light, is transmitted to the C-terminal regulatory domain to initiate the signalling cascade [1], [2]. Consequently, in the present work we have studied this problem by using a range of biophysical techniques. In PELDOR studies of the N-terminal photosensory region of Cph1 we measured a distance of 36 Å between neighboring Cys371 residues in the protein dimer for both the Pr and Pfr states. Cys371 is found on the first strand of the β-sheet of the PHY domain, close to the proposed interface with the HK region, which would place the spin labels more than 65 Å apart in a ‘head-to-tail’ arrangement originally proposed for Cph1 [5]. The PELDOR measurements are, however, compatible with the parallel ‘head-to-head’ structures observed in other phytochromes [7], [23], [28] and it is likely that the previous ‘head-to-tail’ alignment is a result of the crystallization conditions used [5]. The *Ps. aeruginosa* structure suggests a distance of approximately 45 Å between the β-carbons of the equivalent residues, Met358, in the ‘head-to-head’ structure [7]. However, this does not account for the lengths of the spin labels or for the decreased distance between PHY domains suggested by the cryo-EM studies relative to the distance exhibited in crystals [23].

PELDOR measurements on the single-labeled full-length Cph1 protein show that there are considerable differences in the inter-label distance compared to the isolated photosensory region for both the Pr and Pfr states. These data suggest that a conformational equilibrium is established in full-length Cph1 and includes conformations in which the PHY domains are more than 10 Å closer together than the single observed distance observed for the photosensory region. Moreover, this conformational distribution differs in the Pr and Pfr states of the protein, suggesting that there is an overall relative decrease in the distance between neighboring PHY domains upon photoconversion from the Pr form to the Pfr form. The PHY domain is predicted to be the most mobile of the three domains of the photosensory region and forms part of the interface with the HK region [7], [28], which may explain the differences between the full-length protein and the isolated photosensory region. In addition, we have shown that the functional mobility of the PHY domain is dependent on the photoisomerization of the bilin cofactor, which is bound to the adjacent GAF domain. This is supported by studies on the photosensory region of phytochromes that lack the PHY domain, which can no longer stabilize the Pfr state and are unable to undergo photoconversion [5], [29].

**Figure 8:**
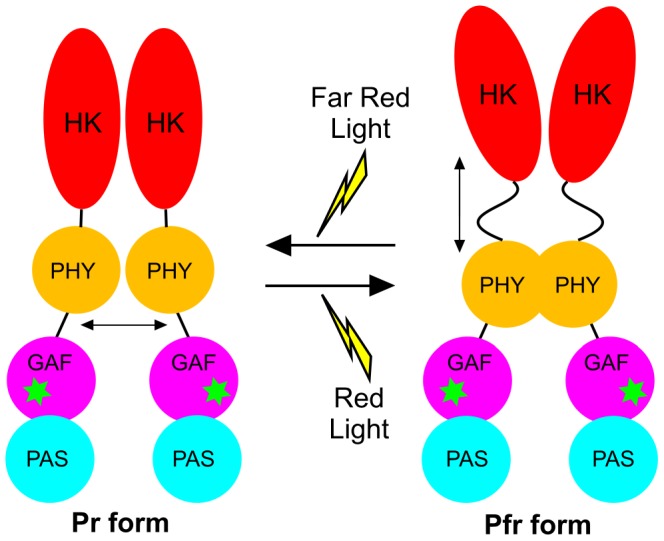
Scheme showing proposed domain movements during photoconversion of the Pr to Pfr forms of Cph1. The asterisks indicate the phycocyanobilin cofactor.

In our double-labeled Cph1 protein the N733 residue, which has been changed to Cys (N733C), is predicted to lie in the first strand of the catalytic and ATP binding (CA) domain of the HK region, in sequence comparisons between phytochromes and HKs of known structure. The CA domain is necessarily mobile about the central helical core of the HK region in order to allow for autophosphorylation within the HK dimer, be it *cis* or *trans* [30], [31]. The PELDOR data show that two additional inter-label distances are introduced with the additional spin label at C733 for both the Pr and Pfr states of the protein, which could be caused by several possible dipolar interactions that are almost impossible to distinguish from each other. However, the known structures of HKs [30], [31] show distances between the equivalent residues to C733 in the HK dimer of over 60 Å, which is too long to be measured in our experiments. Similarly, the distance between C733 and the C371 label on the *trans* PHY domain (i.e. on the other half of the dimer) is also too long to be measured by PELDOR and therefore, prevents the detection of this interaction. Thus, we assign the additional distances to interactions between the spin labels on the PHY and HK-CA domains in the same monomer. Although this may reflect the conformational equilibrium of the PHY domain, it appears unlikely that we are observing interactions between the C733 label (at only 40% occupancy) and one of the minor conformations of the PHY domain. Therefore, the two additional distances measured for each form (21 Å and 19 Å for Pr and 25 Å and 21 Å for Pfr) are assigned to a conformational distribution in the HK-CA domain, which then interacts with the major conformation of the PHY domain. These distances are consistent with the model structures proposed for the full-length bacteriophytochrome [7] and the recent cryo-electron microscopy studies of *D. radiodurans* phytochrome [23]. Consequently, this leads us to propose a schematic model for the inter- and intra-domain movements that are required for light signalling in Cph1 (Figure 8). In this model, following photoconversion from the Pr state to the Pfr state there is a relative decrease in the distances between neighboring PHY domains, whereas the relative distances between the PHY domain and HK-CA domain within the same monomer increases. These conformational changes are likely to be necessary to correctly orientate the HK-CA domain to trigger the signalling cascade upon photoisomerization of the bilin cofactor.

The cryo-trapping and time-resolved measurements provide a description of the protein motions required for each of the individual steps in the Pr → Pfr reaction and suggest that differences exist in the roles of the conformational changes that are coupled to each step. The ultrafast formation of the primary photoproduct, termed the Lumi-R state, involves a *Z*-*E* isomerization of the methine bridge of the bilin chromophore, either between rings C and D or rings A and B. We have used cryogenic absorbance measurements to trap this intermediate at different temperatures and have shown that it can be formed below ∼180 K. However, at approximately 200 K it is known that proteins undergo a dynamic transition, termed the ‘glass transition’, below which any large-scale conformational changes in the protein that require solvent reorganization become frozen out [32]–[40]. At these lower temperatures the only motions that continue are the internal, localized protein motions, which are essentially independent of the bulk solvent fluctuations, such as methyl and aromatic ring rotation [37]–[39]. As formation of the Lumi-R state can still proceed well below 200 K, it demonstrates that large-scale domain movements or reorganization of the protein does not accompany this stage of the photoconversion. In addition, we have found that the initial photoisomerization dynamics, on the picosecond timescale, are independent of solvent viscosity, which confirms that there is a lack of any coupled long-range motions [36]–[40]. Consequently, initial photoisomerization reaction involves structural changes within the bilin molecule itself and protein motions are restricted to only minor localized adjustment around the bilin cofactor [15], [16]–[21].

In contrast to the initial photoisomerization reaction to form the Lumi-R intermediate, the ensuing non-photochemical steps in the formation of the final Pfr state of Cph1 can only occur at much higher temperatures, implying a role for the large-scale protein motions that become frozen out below the ‘glass transition’ temperature [32]–[35], [37], [38]. Moreover, the rates of the first 3 steps that comprise these latter stages of the phytochrome photocycle are very sensitive to changes in the viscosity and the dielectric constant of the solvent, suggesting that they involve a series of solvent-coupled protein motions [36]–[40]. Therefore, it is likely that these final steps of the Pfr formation, which are also coupled to the deprotonation and reprotonation of the chromophore [22], involve the conformational changes and/or domain movements in the PHY and HK-CA domains that are crucial in switching between the active and the inactive state of the phytochrome. Moreover, the lack of any isosbestic point in the cryogenic absorbance measurements suggests that there may be multiple transitions or intermediates, which tends to support the conformational equilibrium that was observed in the PELDOR measurements.

### Concluding Remarks

Cph1 dimers are arranged in a parallel ‘head-to-head’ structure. Photoconversion between the Pr and Pfr forms of phytochrome involves a hierarchy of conformational changes in both the PHY and HK-CA domains of the protein. These conformational changes are driven by the initial ultrafast photoisomerization of the bilin cofactor, where localized structural changes in the GAF domain are subsequently transmitted and amplified to cause large-scale conformational changes in the adjacent PHY and HK-CA domains. Our model of these conformational changes involves neighboring PHY domains moving closer together in conjunction with the PHY and HK-CA domains within the same monomer moving further apart. These transitions occur on the millisecond to second timescale following photoconversion from the Pr state to the Pfr state. These conformational changes orientate the HK-CA domain in an active configuration that allows the signalling cascade to be initiated.

## Materials and Methods

### Protein Preparation

All chemicals were obtained from Sigma-Aldrich unless otherwise stated. Recombinant full-length Cph1 protein from *Synechocystis* sp. PCC6803 was expressed and purified as a phycocyanobilin-bound holoprotein using a dual-plasmid *Escherichia coli* expression system [41]. The gene for Cph1 was synthesized (GenScript) and cloned into the pBAD/HisB expression vector (Invitrogen). The genes encoding the bilin synthetic enzymes, heme oxygenase and phycocyanobilin:ferredoxin oxidoreductase from *Synechocystis* sp. PCC6803, were synthesized (GenScript) and cloned into the dual expression vector pCOLADuet-1 (Novagen). Both plasmids were cloned into *E. coli* strain BL21 and selected for ampicillin and kanamycin resistance. Cells were grown in 800 ml of Luria–Bertani medium to an OD_600_ of ∼0.5 at 20°C and expression of the bilin synthetic genes was induced by the addition of 1 mM isopropyl b-D-thiogalactoside. Cph1 expression was induced by the addition of 0.2% L-arabinose after incubation for 1 hr at 20°C and cells harvested by centrifugation after a further 16 hrs growth at 20°C. Cell lysate in 50 mM Tris-HCl pH 7.5, 500 mM NaCl, 5 mM imidazole, 10% glycerol, 1 mM 2-mercaptoethanol, 0.1% Triton X-100 was applied to a 20 ml Ni^2+^ sepharose (Qiagen) affinity chromatography column, washed with 50 mM imidazole and recombinant protein eluted with 250 mM imidazole. The protein was purified further using a Superdex 200 gel filtration chromatography column (GE Healthcare), pre-equilibrated with 50 mM Tris-HCl pH 7.5, 100 mM NaCl, 10% glycerol 1 mM 2-mercaptoethanol, 0.1% Triton X-100. Samples were concentrated using a Vivaspin 20 spin concentrator (Sartorius) before further studies.

Site-directed mutagenesis was used to produce the N733C variant of Cph1 to replace Asn733 in the C-terminal HK domain by a Cys residue. The primers (MWG Eurofins) used for the mutagenesis were as follows:

Forward primer: 5′-CTGGAAAGCAGCCCGGGTGAAGGCTCTA-3′.

Reverse primer: 5′-GTAGAGCCTTCACCCGGGCTGCTTTCCAG-3′.

The Asn733Cys mutation was verified by DNA sequencing (MWG Eurofins). The N733C protein was expressed and purified as described above.

### Absorbance Spectroscopy

All absorbance spectra were measured using a Cary 50 spectrophotometer (Agilent Technologies). For low temperature measurements 1 mL samples in 44% glycerol and 20% sucrose containing 50 mM Tris-HCl, pH 7.5, 0.1% Genapol, 0.1% 2-mercaptoethanol were cooled to 77 K at an approximate rate of 10 K per minute in an Opstistat DN liquid nitrogen cryostat (Oxford Instruments Inc.) to record spectra. Samples were then warmed to the desired temperature at an approximate rate of 10 K per minute to initiate the reaction, either by illumination with red light for 10 mins or by subsequent incubation in the dark for 10 mins, before cooling again to 77 K to record spectra. Illumination with red light (approx. 1500 µmole m^−2^s^−1^) was provided by a Schott KL1500 electronic cold light source with a band pass filter at 660 nm (Andover Corp) to trigger the Pr to Pfr conversion.

### Transient Pump-probe Spectroscopy

The laser system used for the transient absorption experiments comprises a Ti:sapphire amplifier (a hybrid Coherent Legend Elite-F-HE) pumped by a Q-switched Nd:YLF laser (Positive Light Evolution-30) and seeded by a Ti:sapphire laser (Spectra-Physics Mai Tai). The amplifier output, which has a wavelength of 800 nm, a 1 kHz repetition rate and ∼120 fs pulse duration is split, part of the output is used to pump a non-collinear optical parametric amplifier (Light Conversion TOPAS-White), which produces the pump beam, centred at ca. 580 nm with a FWHM of ca. 50 nm. Another fraction of the amplifier output is used to generate the white light probe pulse for a Helios (Ultrafast Systems LLC) broadband pump-probe transient absorption spectrometer, with an instrument response function of around 170 fs. Data points were collected in a random order over a 350 ps time frame. Samples had an OD at 660 nm of 0.3 and were flowed at a rate of approximately 13 ml/min through a 0.5 mm pathlength quartz cell. The sample reservoir was under constant illumination (approx. 1500 µmole m^−2^s^−1^) from a Schott KL1500 electronic cold light source with a band pass filter at 720 nm to ensure that the protein remained in the Pr form for the duration of the experiment.

### Laser Flash Photolysis

Laser photoexcitation experiments were essentially carried out as previously described [42]. The photoconversion of the Pr state to the Pfr state of Cph 1 was achieved by excitation at 660 nm, using an optical parametric oscillator of a Q-switched Nd-YAG laser (Brilliant B, Quantel) in a cuvette of 1 cm pathlength at 20°C. Laser pulses (∼30 mJ) were between 6–8 ns in duration. Absorption transients were recorded at a range of wavelengths between 600–760 nm using an LKS-60 flash photolysis instrument (Applied Photophysics Ltd) with the detection system (comprising probe light, first monochromator, sample, second monochromator and photomultiplier) at right angles to the incident laser beam. Rate constants were observed from the average of at least three time dependent absorption measurements by fitting to a quadruple exponential function. For viscosity studies, glycerol or sucrose solutions were prepared by weight, and the estimation of their solution viscosity has been previously described [40]. As it is reasonable to assume that the solvent molecules are much smaller than the protein the effect of viscosity on the observed rate of photoconversion can be analyzed by combining the Kramers equation with the Eyring equation to describe the contribution of the protein friction to the total friction of the system according to equation 1 [36], [40]:
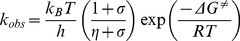
(2)where σ, in units of viscosity, is the contribution of the protein friction and η is the absolute viscosity. Although this equation fails to reflect observed protein behaviour in the ‘overdamped Kramer’s limit’ (high viscosities) as discussed in [43], its application is valid within the range of viscosities employed here.

### PELDOR Spectroscopy

Spin-labeling of Cph1 samples was performed in an anaerobic glovebox (Belle Technology) under a nitrogen atmosphere (oxygen maintained at <2 ppm). Reduced Cph1 protein was dialyzed against 2-fold molar excess of MTSL (1-oxyl-2, 2, 5, 5-tetramethylpyrroline-3-methyl-methanethiosulfonate, Toronto Research Chemicals) at 4°C for 3 hours. The spin-labeled protein was passed through a PD-10 desalting column (Bio-Rad) to remove the unreacted spin label and concentrated to a final concentration of 200 µM in 250 µL using a Vivaspin 20 spin concentrator (Sartorius). Spin-labeled proteins were illuminated (approx. 1500 µmole m^−2^s^−1^) in an EPR tube for 15 minutes at 4°C by a Schott KL1500 electronic cold light source with band pass filters at either 660 nm or 720 nm (Andover Corp) to generate the Pfr or Pfr forms of the protein, respectively and flash frozen in liquid N_2_ prior to EPR experiments. Mass spectral analysis was used to confirm the attachment of the MTSL spin label (see supplementary section).

All EPR spectra were obtained using a Bruker ELEXSYS E500/E580 spectrometer operating at X-band. Temperature control was effected using liquid helium and an Oxford Instruments ESR900, for continuous wave EPR, or CF935, for pulsed measurements, cryostat connected to an ITC503 temperature controller. Continuous wave spectra were obtained at 80 K using the parameters given in the figure caption. Two pulse echo decays [44] were obtained at several temperatures between 80 K and 30 K using a π/2-τ-π-τ-acquire sequence with a 32 ns π pulse length and τ starting at 200 ns using 1024 8 ns increments. The 30 K experiments offered the best compromise between T_M_ and T_1e_ (T_M_ needs to be long enough to allow the echo be detected in the subsequent four pulse ELDOR experiments while T_1e_ must be short enough to allow for relaxation delays of only a few ms) and these data are shown in Figure S1. Pulsed field swept EPR spectra [45] were obtained at 80 K using soft pulses in order to avoid excitation bandwidth-induced spectrum broadening. The π/2-τ-π-τ-acquire pulse sequence was employed, with π = 200 ns and τ = 1 µs. Four pulse ELDOR [24] experiments were recorded at 30 K using a π/2-T-π-T+τ-π-τ-acquire sequence with T = 230 ns and τ = 810 ns and quadrature detection. The π pulse length was 32 ns. The ‘fourth’ pulse, a π pulse applied at the pump microwave frequency, was incremented in 8 ns steps during the T+τ period starting at 100 ns after the second pulse. The interval τ was limited to 810 ns due to the spin-spin relaxation, phase memory time T_M_, of the nitroxide radicals which precluded detection of the refocused echo at longer values of τ without a reduction in temperature that unacceptably lengthened the 2 ms pulse sequence repetition time (500 Hz repetition frequency). The timing of the ‘fourth’ pulsed is indicated as t with t = 0 at 100 ns after the second π pulse and t incrementing by 8 ns for each of 115 data points thereafter. The refocussed echo was phase corrected positive maximum with no contribution to the out of phase (90°) quadrature channel before acquisition. The real component from the raw ELDOR time evolution data was selected (there was invariably no echo and thus no echo modulation, detected in the imaginary channel), left-shifted to create a cosine function (starting at the first maximum), baseline corrected using a third order polynomial and then a Hamming window function was applied in order to increase signal to noise following the subsequent Fourier transform and drive the decay function to completion, thus avoiding truncation effects. Each data set was then zero filled to 1024 point prior to Fourier transformation. We have chosen to adopt the direct conversion approach for the determination of distances from dipolar time evolution data [46] as we are dealing with distances of less than 50 Å and angular correlation between labels is expected to be negligible due to the nature of the linker. We also believe this approach provides the most objective analysis in this case as it requires no input beyond the raw data itself.

## Supporting Information

Figure S1 **Mass spectral analysis of nitroxide-labeled Cph1 at Cys371.** The tryptic peptide LLGLTGSQGAAICFGEK containing Cys371 was the precursor selected for fragmentation. This resulted in a number of fragment ions relating to the precursor ion isolated at m/z 924.98 at retention time 55.34 minutes. Peptide fragments are indicated by b if the charge is retained on the N-terminus or by y if the charge is retained on the C-terminus. The y ions are displayed in blue and the b ions in red. Peaks in black have not been associated with standard ions from this peptide. In this analysis, the y-ions y3 through to y15 have identified peaks, which correspond to the amino acids F through to G on the blue ladder. Consequently this means that the amino acid sequence from y3 through to y15 has been sequenced, which includes the MTSL containing cysteine modification. Additionally, the b ions have been identified for b3 to b6, b8 to b10 and b12 to b14, which also includes the MTSL modified Cys residue at the b12 ion. Taken together, the MTSL modified Cys371 residue for this peptide has been conclusively identified.
(TIF)

Figure S2 **Two pulse echo decay traces obtained from spin-labeled** ***Synechocystis*** **PCC 6803 Cph1.** (A). Pr form of the N-terminal photosensory region with spin-label at C371. (B). Pfr form of the N-terminal photosensory region with spin-label at C371. (C). Pr form of full-length Cph1 with spin-label at C371. (D). Pfr form of full-length Cph1 with spin-label at C371. (E). Pr form of full-length Cph1 with spin-label at C371 and N733C. (F). Pfr form of full-length Cph1 with spin-label at C371 and N733C. Pulse sequences and data processing are described in Materials and Methods.
(TIF)

Figure S3 **Two pulse echo-detected field sweep traces obtained from spin-labeled** ***Synechocystis*** **PCC 6803 Cph1.** (A). Pr form of the N-terminal photosensory region with spin-label at C371. (B). Pfr form of the N-terminal photosensory region with spin-label at C371. (C). Pr form of full-length Cph1 with spin-label at C371. (D). Pfr form of full-length Cph1 with spin-label at C371. (E). Pr form of full-length Cph1 with spin-label at C371 and N733C. (F). Pfr form of full-length Cph1 with spin-label at C371 and N733C. Pulse sequences and data processing are described in Materials and Methods.
(TIF)

Figure S4 **Baseline corrected four-pulse ELDOR traces.** Baseline corrected four-pulse ELDOR traces were obtained from spin-labeled *Synechocystis* PCC 6803 Cph1 produced by subtraction of a third order polynomial from the raw data shown in the left hand panel of Figure 1. (A). Pr form of the N-terminal photosensory region with spin-label at C371. (B). Pfr form of the N-terminal photosensory region with spin-label at C371. (C). Pr form of full-length Cph1 with spin-label at C371. (D). Pfr form of full-length Cph1 with spin-label at C371. (E). Pr form of full-length Cph1 with spin-label at C371 and N733C. (F). Pfr form of full-length Cph1 with spin-label at C371 and N733C. Pulse sequences and data processing are described in Materials and Methods.
(TIF)

Figure S5 **Absorbance spectra of Cph1 at low temperatures following illumination.** Samples contained 15 µM Cph1 and this was illumination for 10 mins at different temperatures ranging from 77 K to 180 K. The formation of the absorbance peak at 684 nm and simultaneous disappearance of the absorbance band at 668 nm at higher temperatures are indicated by the arrows. (B). 77 K absorbance spectra of samples containing 15 µM Cph1 after illumination at 180 K for 10 mins and incubation in the dark for 10 mins at increasing temperatures. The arrows indicate the formation and disappearance of the different absorbance bands at higher temperatures.
(TIF)

Figure S6 **The temperature dependence of the steps involved in the Pr → Pfr photoconversion.** (A). The temperature dependence of the initial formation of the Lumi-R state was measured by plotting out the intensity of the absorbance band at 684 nm against the temperature of illumination. (B). The temperature dependence of the remaining step(s) to form the Pfr state was measured by plotting out the intensity of the absorbance band at 715 nm against the temperature of incubation in the dark after illumination with red light at 180 K for 10 mins.
(TIF)

Figure S7 **Kinetic traces observed after photoexcitation.** Typical kinetic traces measured at 660 nm over 1 second and 0.5 ms (inset) following photoexcitation of 15 µM Cph1 (Pr form) with a 6 ns laser pulse at 660 nm. Transients were collected at 20°C as described in the Materials and Methods section.
(TIF)

Figure S8 **Rate constant dependence on glycerol concentration.** The dependence of the rate constant on the concentration of glycerol for the 1^st^ (**A**), 2^nd^ (**B**), 3^rd^ (**C**) and 4^th^ steps (**D**) of the increase in absorbance at 720 nm are shown. All measurements were recorded over a range of timescales as described in the Materials and Methods. The error bars were calculated from the average of at least 3 traces.
(TIF)

Methods S1 **Mass spectrometry analysis.**
(DOC)
