# Supplementary figures and images for: Ultrafast Red Light Activation of Synechocystis Phytochrome Cph1 Triggers Major Structural Change to Form the Pfr Signalling-Competent State
Source: PLoS One. 2012 Dec 26;7(12):e52418. doi: 10.1371/journal.pone.0052418 (PMC3530517; doi:10.1371/journal.pone.0052418)

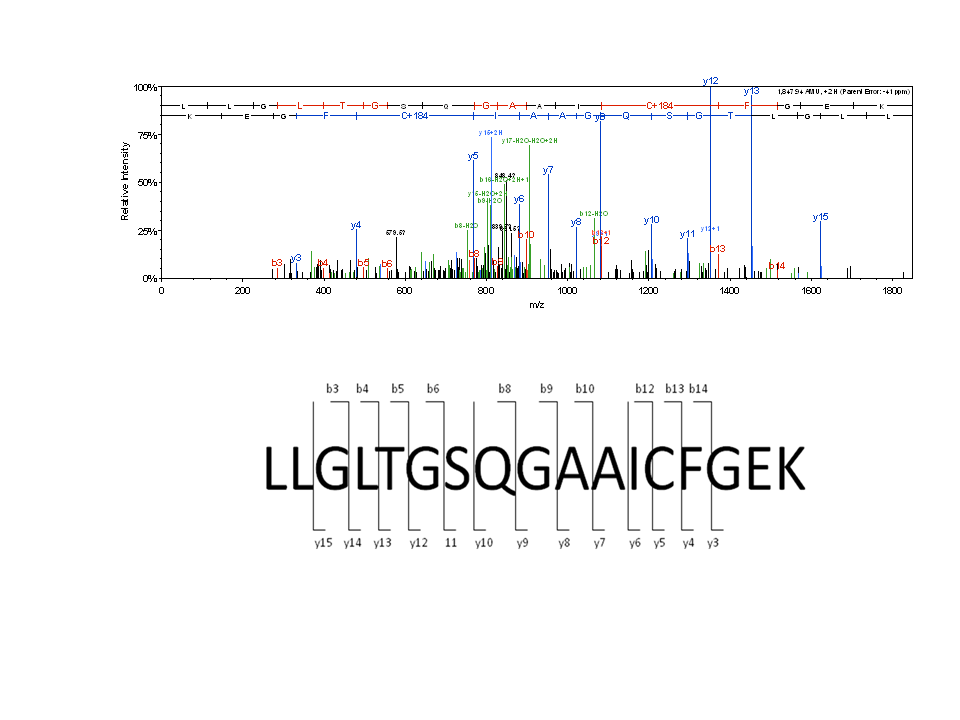

Supplement: Figure S1 — Mass spectral analysis of nitroxide-labeled Cph1 at Cys371. The tryptic peptide LLGLTGSQGAAICFGEK containing Cys371 was the precursor selected for fragmentation. This resulted in a number of fragment ions relating to the precursor ion isolated at m/z 924.98 at retention time 55.34 minutes. Peptide fragments are indicated by b if the charge is retained on the N-terminus or by y if the charge is retained on the C-terminus. The y ions are displayed in blue and the b ions in red. Peaks in black have not been associated with standard ions from this peptide. In this analysis, the y-ions y3 through to y15 have identified peaks, which correspond to the amino acids F through to G on the blue ladder. Consequently this means that the amino acid sequence from y3 through to y15 has been sequenced, which includes the MTSL containing cysteine modification. Additionally, the b ions have been identified for b3 to b6, b8 to b10 and b12 to b14, which also includes the MTSL modified Cys residue at the b12 ion. Taken together, the MTSL modified Cys371 residue for this peptide has been conclusively identified. (TIF) [file pone.0052418.s001.tif]

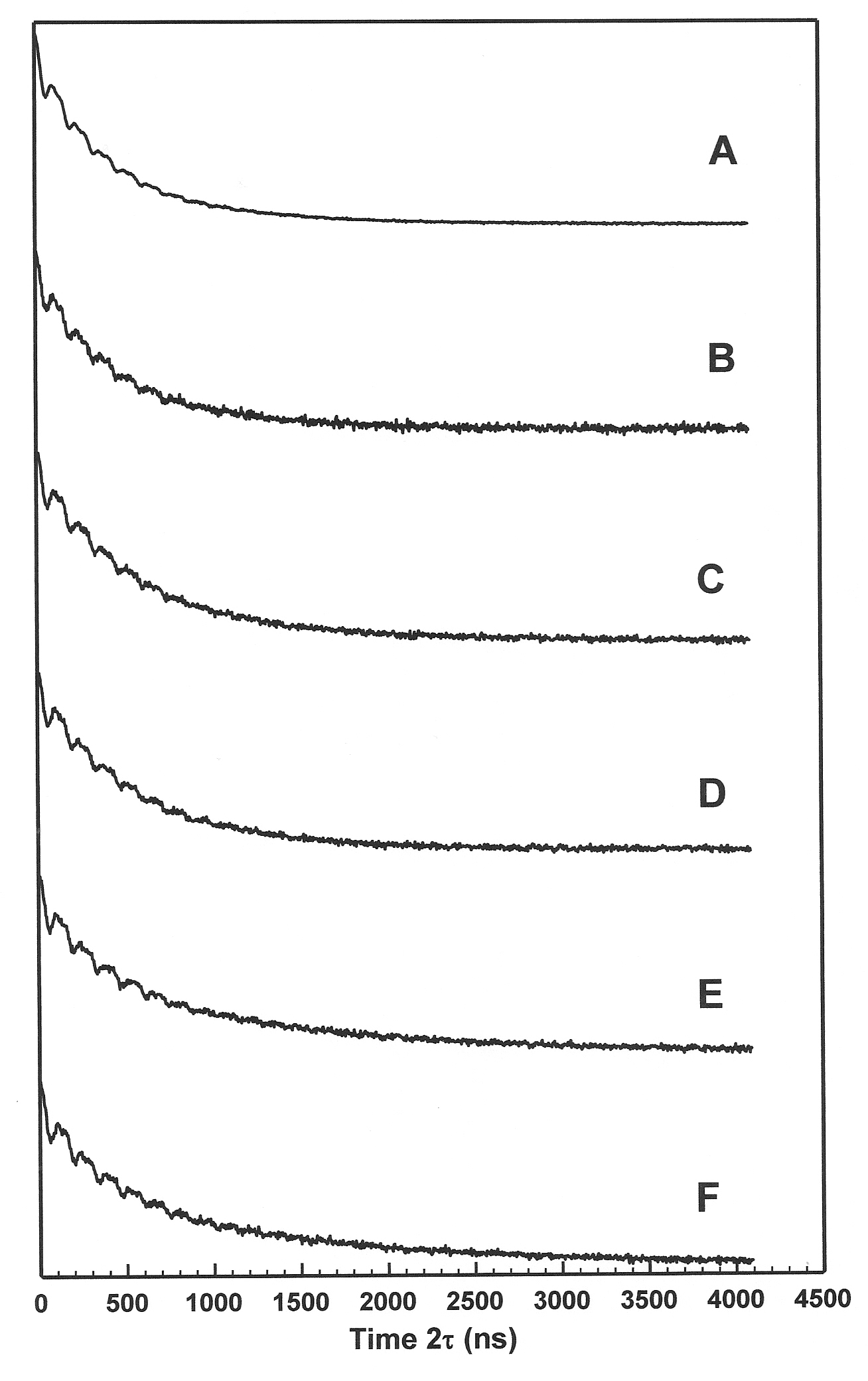

Supplement: Figure S2 — Two pulse echo decay traces obtained from spin-labeled Synechocystis PCC 6803 Cph1. (A). Pr form of the N-terminal photosensory region with spin-label at C371. (B). Pfr form of the N-terminal photosensory region with spin-label at C371. (C). Pr form of full-length Cph1 with spin-label at C371. (D). Pfr form of full-length Cph1 with spin-label at C371. (E). Pr form of full-length Cph1 with spin-label at C371 and N733C. (F). Pfr form of full-length Cph1 with spin-label at C371 and N733C. Pulse sequences and data processing are described in Materials and Methods. (TIF) [file pone.0052418.s002.tif]

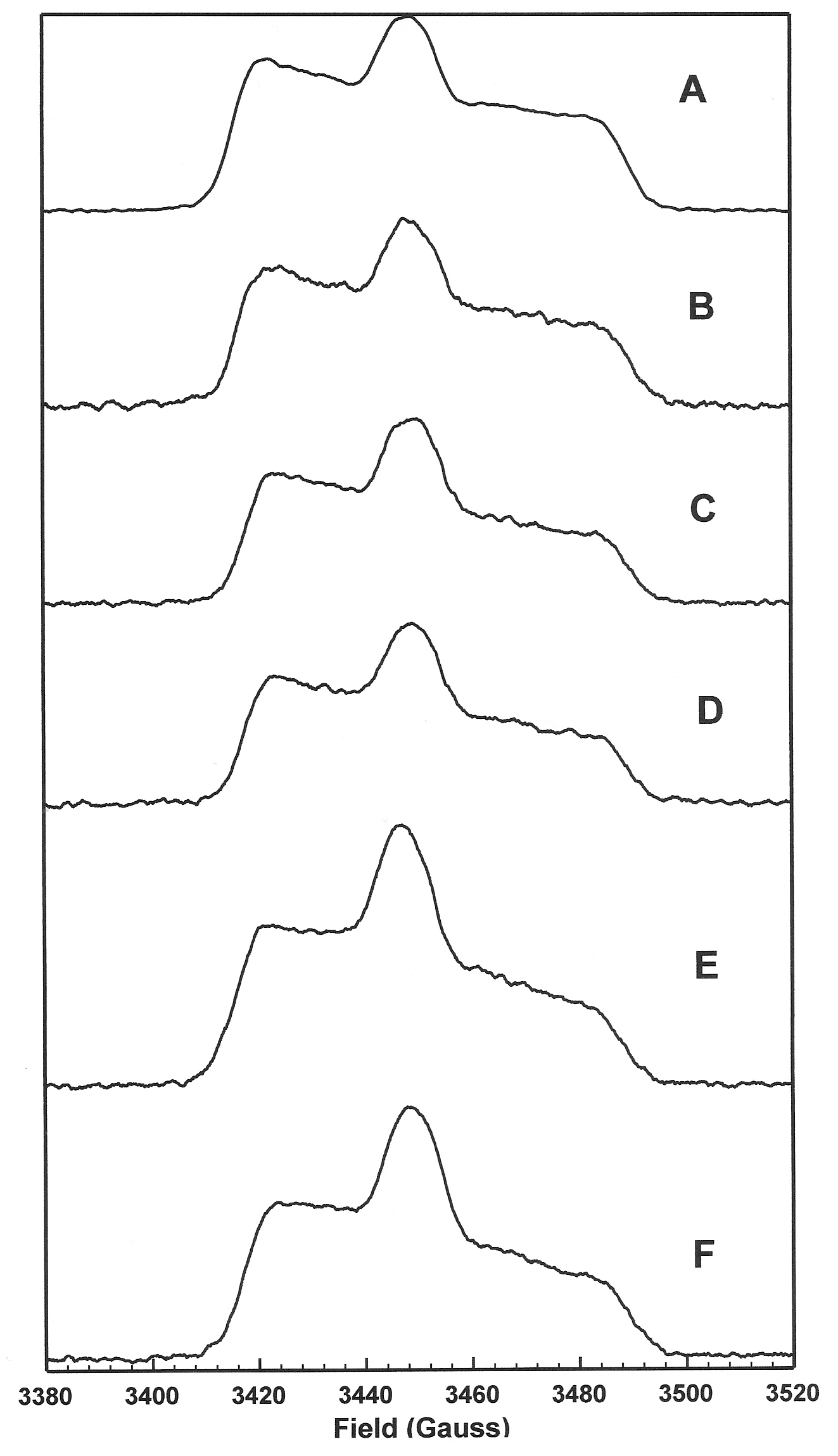

Supplement: Figure S3 — Two pulse echo-detected field sweep traces obtained from spin-labeled Synechocystis PCC 6803 Cph1. (A). Pr form of the N-terminal photosensory region with spin-label at C371. (B). Pfr form of the N-terminal photosensory region with spin-label at C371. (C). Pr form of full-length Cph1 with spin-label at C371. (D). Pfr form of full-length Cph1 with spin-label at C371. (E). Pr form of full-length Cph1 with spin-label at C371 and N733C. (F). Pfr form of full-length Cph1 with spin-label at C371 and N733C. Pulse sequences and data processing are described in Materials and Methods. (TIF) [file pone.0052418.s003.tif]

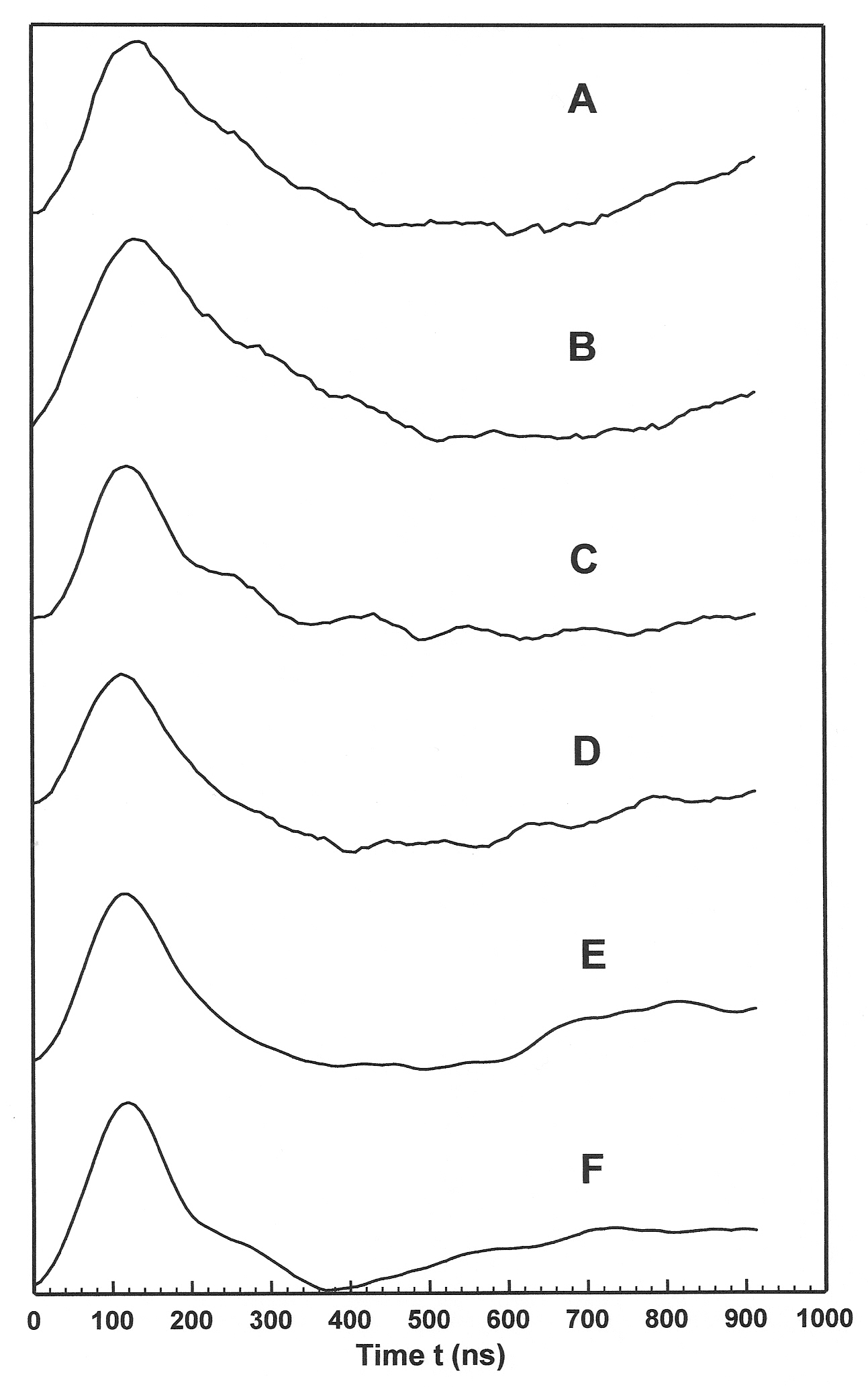

Supplement: Figure S4 — Baseline corrected four-pulse ELDOR traces. Baseline corrected four-pulse ELDOR traces were obtained from spin-labeled Synechocystis PCC 6803 Cph1 produced by subtraction of a third order polynomial from the raw data shown in the left hand panel of Figure 1. (A). Pr form of the N-terminal photosensory region with spin-label at C371. (B). Pfr form of the N-terminal photosensory region with spin-label at C371. (C). Pr form of full-length Cph1 with spin-label at C371. (D). Pfr form of full-length Cph1 with spin-label at C371. (E). Pr form of full-length Cph1 with spin-label at C371 and N733C. (F). Pfr form of full-length Cph1 with spin-label at C371 and N733C. Pulse sequences and data processing are described in Materials and Methods. (TIF) [file pone.0052418.s004.tif]

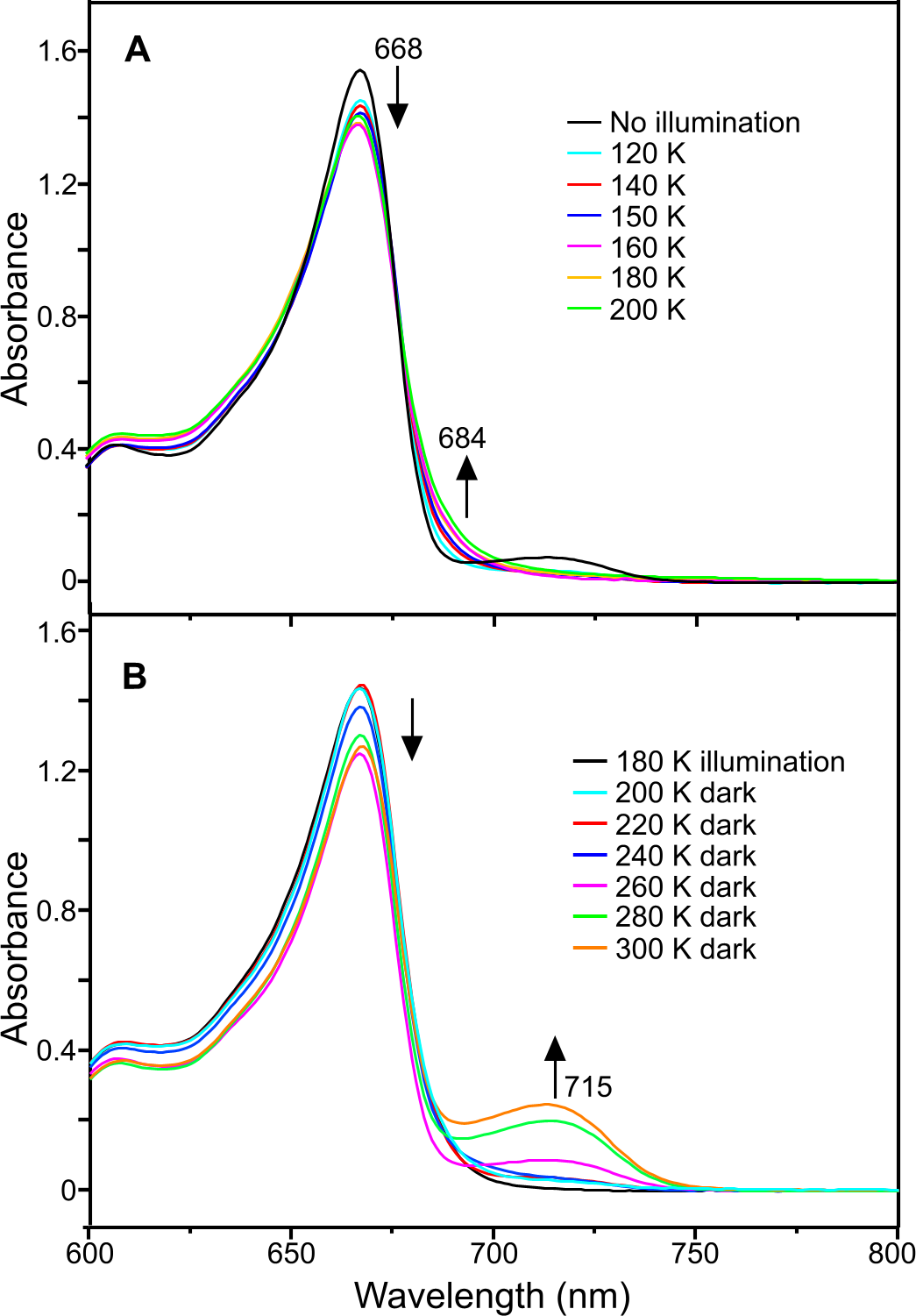

Supplement: Figure S5 — Absorbance spectra of Cph1 at low temperatures following illumination. Samples contained 15 µM Cph1 and this was illumination for 10 mins at different temperatures ranging from 77 K to 180 K. The formation of the absorbance peak at 684 nm and simultaneous disappearance of the absorbance band at 668 nm at higher temperatures are indicated by the arrows. (B). 77 K absorbance spectra of samples containing 15 µM Cph1 after illumination at 180 K for 10 mins and incubation in the dark for 10 mins at increasing temperatures. The arrows indicate the formation and disappearance of the different absorbance bands at higher temperatures. (TIF) [file pone.0052418.s005.tif]

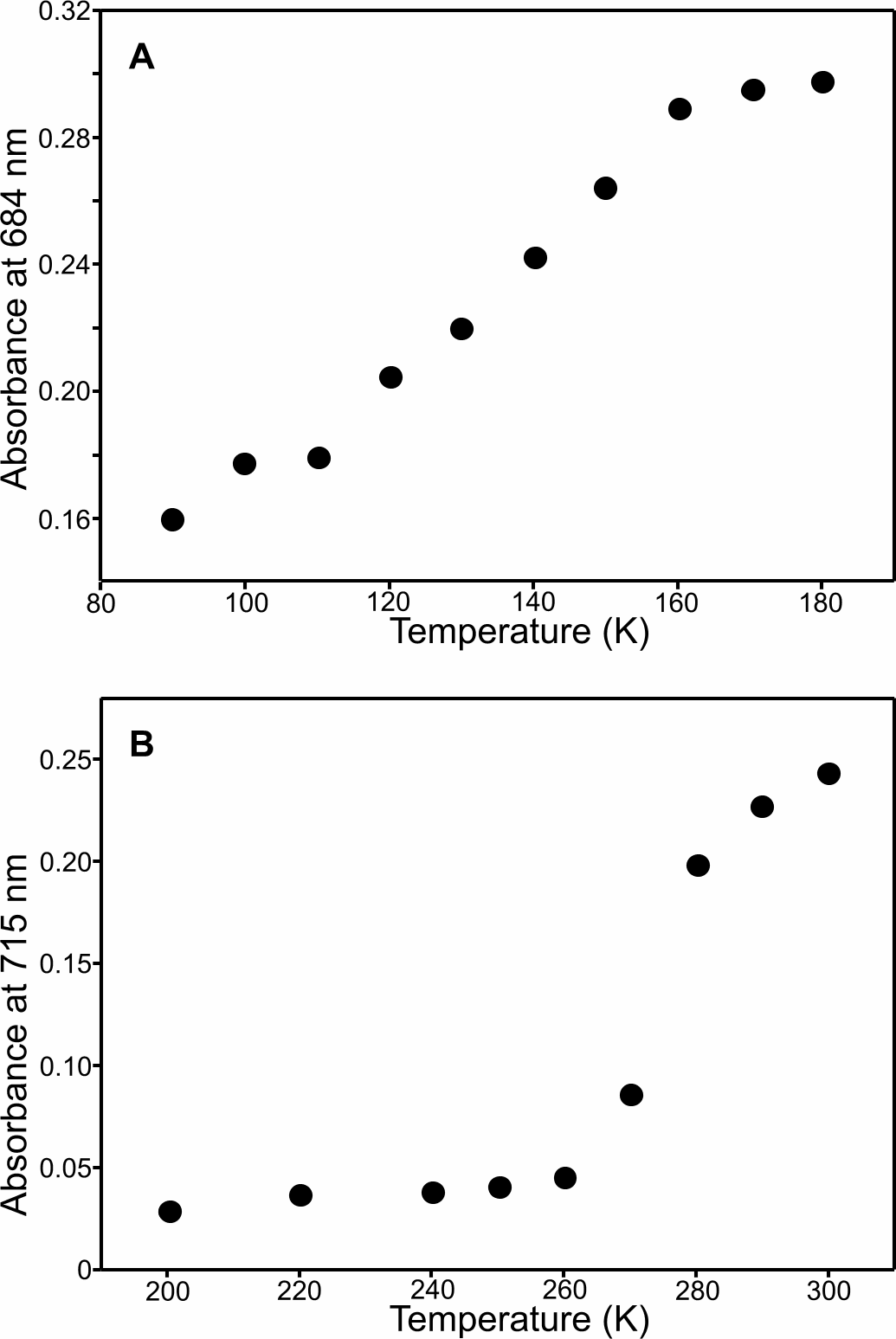

Supplement: Figure S6 — The temperature dependence of the steps involved in the Pr → Pfr photoconversion. (A). The temperature dependence of the initial formation of the Lumi-R state was measured by plotting out the intensity of the absorbance band at 684 nm against the temperature of illumination. (B). The temperature dependence of the remaining step(s) to form the Pfr state was measured by plotting out the intensity of the absorbance band at 715 nm against the temperature of incubation in the dark after illumination with red light at 180 K for 10 mins. (TIF) [file pone.0052418.s006.tif]

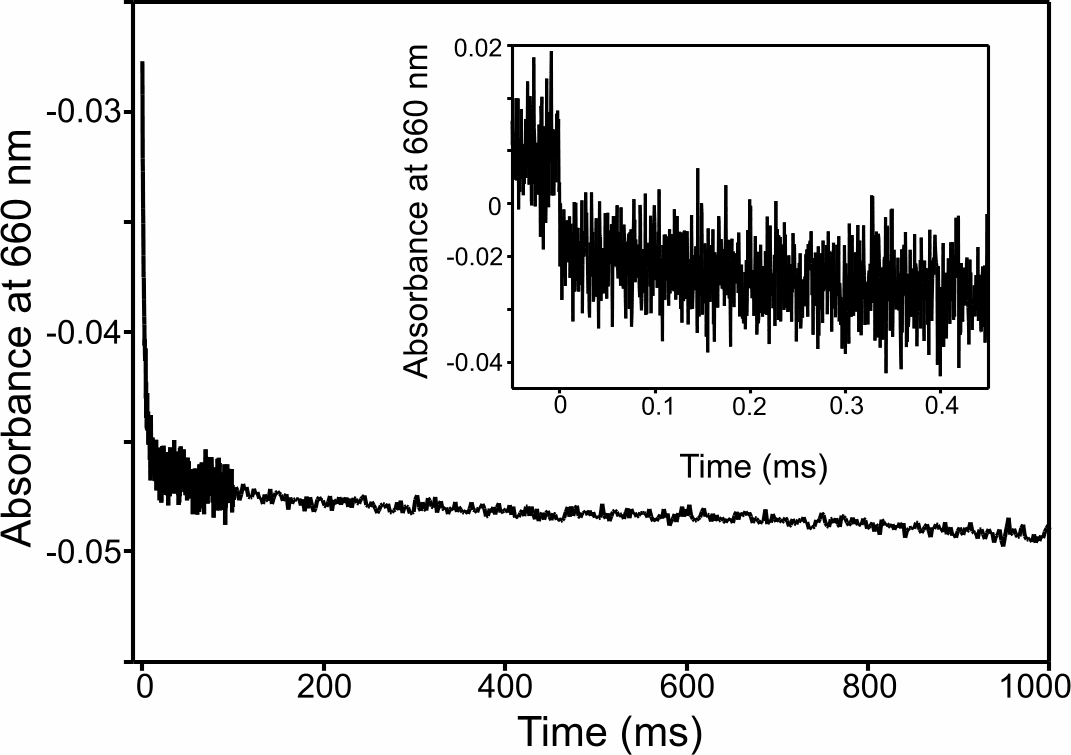

Supplement: Figure S7 — Kinetic traces observed after photoexcitation. Typical kinetic traces measured at 660 nm over 1 second and 0.5 ms (inset) following photoexcitation of 15 µM Cph1 (Pr form) with a 6 ns laser pulse at 660 nm. Transients were collected at 20°C as described in the Materials and Methods section. (TIF) [file pone.0052418.s007.tif]

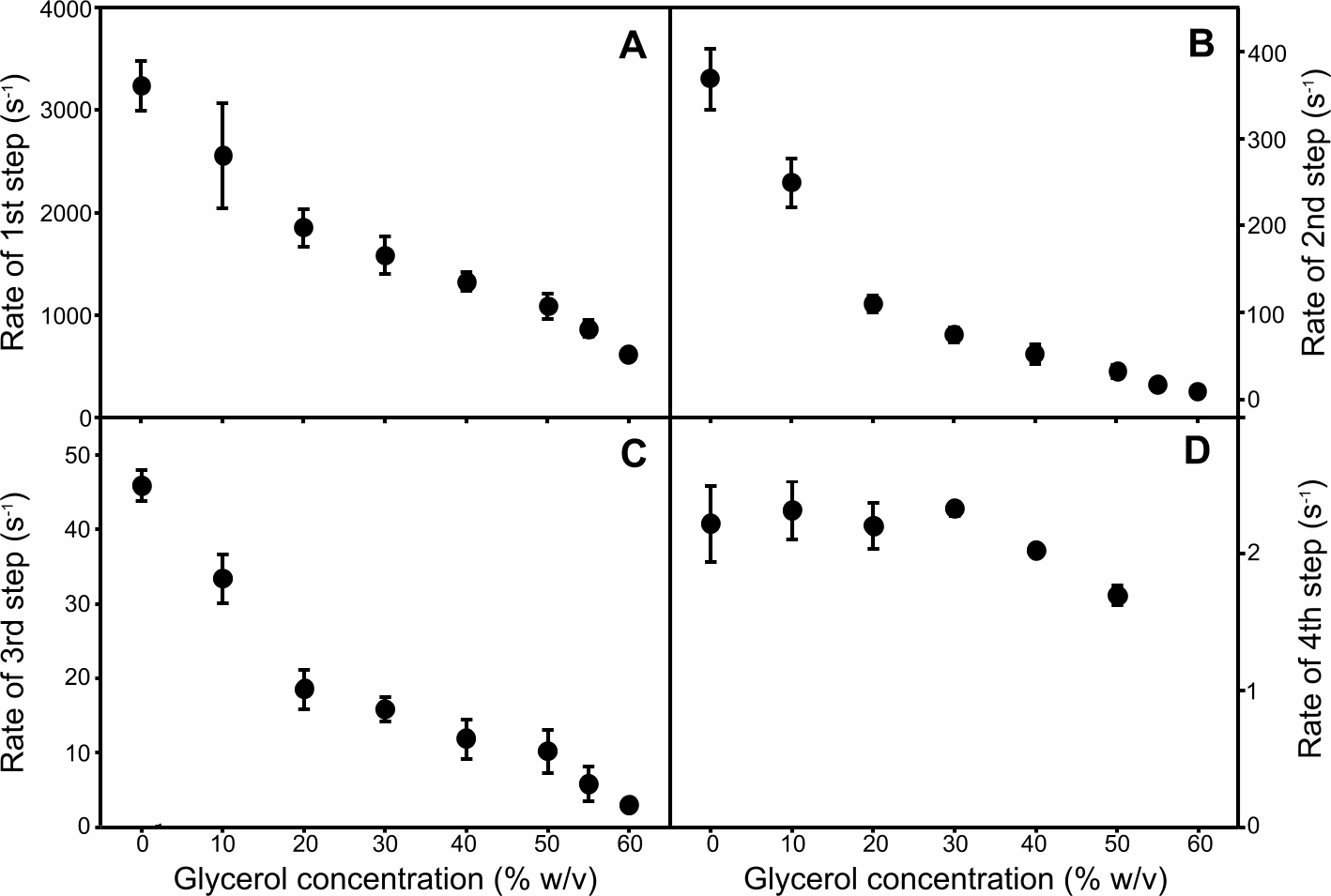

Supplement: Figure S8 — Rate constant dependence on glycerol concentration. The dependence of the rate constant on the concentration of glycerol for the 1st (A), 2nd (B), 3rd (C) and 4th steps (D) of the increase in absorbance at 720 nm are shown. All measurements were recorded over a range of timescales as described in the Materials and Methods. The error bars were calculated from the average of at least 3 traces. (TIF) [file pone.0052418.s008.tif]
