# Supplementary material for: Ultrafast Red Light Activation of Synechocystis Phytochrome Cph1 Triggers Major Structural Change to Form the Pfr Signalling-Competent State
Source: PLoS One. 2012 Dec 26;7(12):e52418. doi: 10.1371/journal.pone.0052418 (PMC3530517; doi:10.1371/journal.pone.0052418)
Supplement: Methods S1 — Mass spectrometry analysis. (DOC) [file pone.0052418.s009.doc]

**On-line Supporting Information**

## Ultrafast red light activation of the bilin cofactor in *Synechocystis* phytochrome Cph1 triggers major structural change to form the Pfr signalling-competent state.

Derren J. Heyes, Basile Khara, Michiyo Sakuma, Samantha J. O. Hardman, Ronan O'Cualain, Stephen E. J. Rigby, Nigel S. Scrutton

*Manchester Institute of Biotechnology and Photon Science Institute, Faculty of Life Sciences, University of Manchester, 131 Princess Street, Manchester M1 7DN, UK*

**Supplement to Materials and Methods**

**Mass spectrometry analysis.**

**SDS-polyacrylamide gel electrophoresis of Cph1 protein and protein digestion.**

Cph1 protein (5 µL) was added to 15 µL of deionised water and 5µL of sample buffer (60 mM Tris-HCl pH 6.8, 25 % glycerol, 2 % SDS, 14.4 mM 2-mercaptoethanol, 0.1% bromophenol blue) and boiled for 5 minutes at 95 °C. The sample was cooled and loaded onto a 10 % SDS-PAGE gel and a voltage of 200 V was applied. After 5 minutes the sample had entered the gel and the voltage was stopped. The gel was stained overnight with colloidal Coomassie blue G250 (Sigma). The band was then excised and dehydrated using acetonitrile followed by vacuum centrifugation. Gel pieces were washed alternately with 25 mM ammonium bicarbonate followed by acetonitrile. This was repeated until the pieces were colorless, and the gel pieces dried by vacuum centrifugation. Samples were digested with a combination of trypsin and endoproteinase glu-C (both Sigma-Aldrich) overnight at 37 °C. The protein:enzyme ratio for trypsin and glu-C digestions were 1:50 and 1:30 respectively. Peptides were desalted using Poros R3 beads (Applied Biosystems) with elution in 3 cycles of 50 % acetonitrile. The peptides were dried in a vacuum centrifuge and resuspended in 10 µL of 5 % acetonitrile / 0.1 % formic acid.

Digested samples were diluted 1:200 prior to MS acquisition and analysed by LC-MS/MS using an UltiMate® 3000 Rapid Separation LC (RSLC, Dionex Corporation, Sunnyvale, CA) coupled to either an a LTQ Velos Pro or Orbitrap elite (Thermo Fisher Scientific, Waltham, MA) mass spectrometer. Peptides were separated using a gradient from 99% A (0.1 % formic acid in water) and 1 % B (0.1 % formic acid in acetonitrile) to 25 % B, in 20 min at 200 nL min^-1^, using a 75 mm x 250 μm i.d. 1.7 µM BEH C18, analytical column (Waters). Peptides were selected for fragmentation automatically by data dependant analysis.

MS data were analysed using Mascot (Matrix Science UK) software against an in-house database that contained the Cph1 sequence. Methionine oxidation and MTSL addition were selected as variable modifications. Data were validated using Scaffold (Proteome Software, Portland, OR) and then manually validated using Xcalibur Qual browser v2.1 (Thermo Fisher Scientific, Waltham, MA).
